# A micro credential for interoperability

**DOI:** 10.12688/openreseurope.14083.1

**Published:** 2021-09-15

**Authors:** Pamela Hussey, Subhashis Das

**Affiliations:** 1 CeIC, ADAPT, School of Computing, Dublin City University, Dublin, Dublin, D09W6Y4, Ireland

**Keywords:** Digital Health, Health Informatics, Healthcare Standards, Interoperability, Learning object, Open Science, Open Innovation, Participatory design.

## Abstract

In the midst of a global pandemic the need for health and social care providers to commit to, and deliver on, integrated patient-centered care services has been accelerated. Globally, health and social care programme administrators are turning to digital devices and applications to provide supporting infrastructure which can offer safe access to health information at the point of care. Digitalisation is increasingly considered a key requirement to support diagnostics and therapeutic care services in health care delivery. The open source community are responding to this need to advance integrated care and digital services by providing targeted resources to address the interoperability challenge. Addressing interoperability in health systems is a core part of achieving sustainable enterprise wide integrated care. Using Open Innovation 2.0 methods for advancing knowledge on interoperability, this paper describes the development of a micro credential for knowledge transfer on interoperability created by the Centre for eIntegrated Care (CeIC). Designed and developed to signpost interested stakeholders to targeted material and build understanding and capacity on the topic. The design approach and initial resource content are explained through the lens of a specific research project funded by an Elite S Fellowship to advance leadership and standardisation for Information and Communications Technology (ICT) in Europe.

## Plain language summary

In order for you to benefit from smart devices such as your mobile phone or an electronic health record, information needs to be able to flow across systems that you use, be available and accurate. If you want to share this information with a health care provider it also needs to be portable to transfer accurately to support you and your health care provider’s choices. The European Union are working with industry and public service providers to assist in guiding best approaches to make this happen. Guiding this work are approved EU standards.

We are a research team who are working in Dublin City University in the *Center for eIntegrated Care* (CeIC). We are building resources to educate people interested in advancing the standards in real world projects. The goal is to move the standards along so we can enjoy safe and secure sharing of health information. This paper provides a summary of an online course we have created to do this. It describes our approach to development of resources and how we selected material for the resource. It also provides pictures of the resource that we will be using in a course called a micro credential.

## 1 Introduction

Globally, the education and training of health and social care service providers to deliver planned eHealth and digital services is widely acknowledged. The potential role of digital health technologies in improving public health, and the need to advance health data standardization as part of eHealth systems and services since 2013, has led to a number of targeted policy actions ^1– 5^. More recently the need for targeted training through the development of micro credentials (MC) has also been critiqued and pilot programmes in the context of Ireland are reported as important to progress ^6^. The nexus of domain expertise on the design for new models of care with computer and data scientists is a critical link in order to optimise co-operation and deliver on the anticipated value proposition. Form must follow function in order to ensure that any national planned digital transformation is well aligned to the service providers needs to advance value based healthcare ^7– 9^.

Across a wide range of domains across at university level, the role of academia in addressing how public service needs are best served is under review. In order to address the practical concerns of everyday routines for citizens and society in general, European Union (EU) policy recommends universities provide focused scholarship on research and teaching activities to advance implementation science. Contemporary actions on EU digital targets for 2030 are focusing on knowledge translation and empowering people to modernise public services. Underpinning this policy agenda are national recovery and resilience plans which look to agile co-creation initiatives which can scale up and spread skills and provide long term impact on infrastructure government and business ^9, 10^. Responding to the call, universities within the EU are therefore exploring a new educational pedagogy. New approaches include leveraging activities education, and research, to advance alliances and cross fertilisation across these respective fields.

This paper describes one such initiative which focuses on the creation of a micro credential (MC) to transfer and act as a primer to advance integrated care. A MC can be described as a purposefully designed smart educational pathway which exploits the pedagogical affordances of new digital technologies underpinned with emerging educational models ^11^.

Using Open Innovation 2.0 (OI2.0) ^1^ methods this paper describes a MC for interoperability ^12^. Consistent with OI2.0 approaches, the authors engaged with an established network of stakeholders including university, industry, and government-public service providers to consider specific requirements to provide targeted resources based on requirements to contribute to value based healthcare ^9, 13^. As part of a collaborative engagement through the European Consortium of Innovative Universities (ECIU) and an Elite S Marie Curie fellowship ^14^, the authors used existing research in progress to create resources to support the development of a MC to explain interoperability in context.

**Why micro credentials focused on interoperability?**

A critical driver to support universal health care globally is access to, and development of, sustainable and high quality infrastructure in the form of clean data. Clean data could be described as a process to address data that is duplicated or incorrectly formatted in a data set which can impede interoperability when combining different data sources. This leads to a common challenge in data reporting which is fragmentation of data called heterogeneous data (i.e. semantic heterogeneity) and so the data must be cleaned before being fit for national reporting and analysis ^15, 16^. The progression of data warehouses and data lakes in the past ten years has also placed demands on how to access, package, and structure data to fulfil the anticipated demands for cross domain co-operation of information, to enlighten businesses and address policy agendas. Supporting data collection, storage and analysis, is the need for robust integrated care systems which facilitate shared access to health records clinically. In addition for business and policy use of big data, there is a need for structured data, domain specific information models and schema designed for regional and national analysis and reporting ^8^.

Understanding and advancing standards based approaches for interoperability is also timely with the advancement of artificial intelligence (AI) and machine learning (ML) in the healthcare domain ^16^. For accurate and quality data to be available for use in future policy translation and knowledge production, heterogeneity of data types will also need to be addressed ^17, 18^. As electronic records replace traditional paper charts, and big data and AI advance, so too the challenge associated with change and transformational processes will become more evident. Even though such tools offer many potential benefits for healthcare, such as increased productivity, cost and medical error reduction, if executed poorly the complexity and risk of data breaches increases in addition to the risk of malicious privacy violations ^19^. Through research and knowledge production universities can contribute recommending and providing standards based practical solutions to tackle these challenges in order to progress digital health uptake and use with targeted educational resources ^11, 20, 21^.

The scenarios listed above relating to data quality and data heterogeneity will require interoperability as a key factor influencing delivery of digital health transformation, albeit for different context of use. Despite this identified need, for almost two decades authors report on the prevailing struggles encountered with achieving interoperability at the local and enterprise level ^15^. A research centre in Dublin City University, Ireland entitled the *Centre for eIntegrated Care* (CeIC) ^22^ is focused on devising learning resources to tackle the interoperability challenge. Motives fuelling this interoperability challenge are well documented ^8, 17, 23^, and so as part of our strategic plan for 2021, the authors targeted solutions to contribute in addressing this ongoing challenge. The resources created to support the MC can be accessed via link https://www.ceic.ie with password *sandpitbeta*.

## 2 Methods

This research got ethical approval from Dublin City University (DCU) ethical committee with reference number *DCUREC/2020/217* on 9th November 2020.

### 2.1 Scoping requirements

Based on experience in the field from both computer science and health care provision and using insight from the literature reviewed and expert opinions the authors set about considering how to design targeted resources to inform the development of a MC for interoperability. Considering what the key barriers are on advancing interoperability, the conclusion was that one of the main impacts relates to the vast amount of literature published on the topic ^1, 7, 15^. The breadth and depth of literature relating to the levels of engagement on interoperability which an individual is required to navigate, focus, and subsequently assimilate for learning about the theory and practice of achieving interoperability is significant. In many cases it can lead to information overload. Increasingly domain experts with a background in clinical service delivery are charged with contributing (or in some cases leading) requirements design programmes. In addition such individuals may also contribute to public procurement processes often being appointed to senior executive roles for deployment of integrated care systems. Yet, such stakeholders have limited protected time to learn. Health care professionals are considered vulnerable populations due to contemporary strains on service delivery culminating in to a shortfall in skill mix, adequate work force resourcing in addition to the mounting pressure of the global coronavirus disease 2019 (COVID-19) pandemic ^24^. Protected time by clinical leads to review a dedicated resource designed to understand interoperability in health care in context may yield less fragmented systems and services in the longer term and could support the process in the following way:

- Accelerate the breadth of learning on the topic of interoperability.
- Offer targeted resources for asynchronous learning.
- Connect participants with an interest in the topic engaged in public service and procurement related activity.

### 2.2 Method

Following the initial scoping exercise and using Open Innovation 2.0 (OI2.0) methodology the *Centre for eIntegrated Care* (CeIC) used key attributes of the OI2.0 methodology in the following way:

- Engaged in a Quadruple helix ^2^ approach using focused discussion with a wider stakeholder group such as the European Consortium of Innovative Universities (ECIU), academic institutions, industry partners and public service providers which contributed to identifying key resources for advancement of the planned MC.
- Defined a dedicated shared platform to promote existing open source seminal work to disseminate state of theart resources for advancing interoperability. Creating an online platform is considered a key pillar in the OI 2.0 methodology. This online platform offered a space to share content, promote innovation and match participantsinterests facilitating the potential for development of a shared community ^12, 13^.
- The platform was designed as part of the MC and provided knowledge synthesis from three distinct viewpoints; health and social care domain, computer science and health informatics. Each of the viewpoints provided scaffolds for learning about interoperability and used open science, state of the art resources and selected standards from (International Standards Organisation Technical Committee 215 (ISO TC 215) ^25^ and the European Committee for Standardization Technical Committee (CEN TC 251) ^26^.
- In addition, a Marie Curie Fellowship EliteS, sponsored by Science Foundation Ireland (SFI) and the Adapt Research Centre provided standards based demonstrators ^14^ from research project entitled the Common Semantic Data Model (CSDM) ^27^.

### 2.3 Key findings on requirements identification

***2.3.1 Phase one.*** Key findings from this phase of research activity suggested that for many individuals the need to grasp the topic ofinteroperability was considered challenging but important to their practice. Individuals consulted as part of the processof engagement found the topic confusing and difficult to grasp over a short timeline.

The identified skills and learning outcomes to underpin the development of the MC included:

1. Proficiency in formulating and demonstrating an understanding on the topic of interoperability in the context of a domain specific application and simulated system for health and social care.
2. An ability to define, organise, and build a demonstrator Use Case using recommended EU approaches
3. Foundation expertise on the use, interpretation and application of online tooling to illustrate a detailed sequence and set of related processes to structure data for interoperability.
4. Acquired knowledge and competency to investigate a specific data set to interpret an information schema and ontology

Configurations and decisions of the design approach which informed the learning resources included:

- Key features for effective design for usability such as explanatory material and podcasts.
- Individual sections to support content and flow of the material to be accessed and reviewed as either stand alone or in sequenced manner to encourage asynchronous and iterative learning.
- Decisions on what the key supporting components were to explain the interoperability challenge through a clinical practice lens led to scenarios of context specific cases with additional supporting theory and material. Providing examples of cases and context specific configurations potentially could assist in linking the technology with problems that were based on real world scenarios of practice. Additional queries included how best to represent embedded guidance documents and what information presented was static and what was dynamic. This would have implications for sustainability of the material as regular revision and updating of the resource. A set of discrete learning objects were conceived, mapped, and then devised with dedicated links to associated tooling from the web. Learning objects are described by Littlejohn *et al*. in 2008 as having four discrete levels which include a) digital assets often containing a single file or raw media asset, b) information objects c) learning activities and d) learning design structured sequences of information and activities ^28^.
- Supporting material included a glossary for key terms, not only to understand the variety and mix of concepts and terms related to the subject but also to explain the collection of buzz words and acronyms inherent in the subject matter.

***2.3.2 Phase two.*** To confirm the initial design features were in line with expectations and strategically aligned with the overall design scope, the initial prototype material to support the MC was shared with identified key stakeholder groups to formatively review and advise on the relevance and usefulness of the resources created to support the MC. The key stakeholder group identified to formatively review the prototype material included; small and medium-sized enterprises (SME), industry partners, senior health care professionals with an interest in learning about interoperability, academic colleagues from other universities in Ireland and the USA, in addition to post graduate students completing an MSc in Digital Transformation. Initial collaborative feedback from this group suggested the learning resources were useful and met the identified scope of the design brief, recommending some additional supporting introductory podcasts which were subsequently added to the resource.

Once the initial learning resources were created the Micro Credential (MC) was then submitted and approved at the university committee level. The remainder of this paper detail the supporting resources for the MC now available to access via the centres website ^22^. The source code can be downloaded from Zenodo ^29^.

## 3 Initial development programme

### 3.1 Learning resources to support micro credential (MC) for interoperability

**Figure 1.**
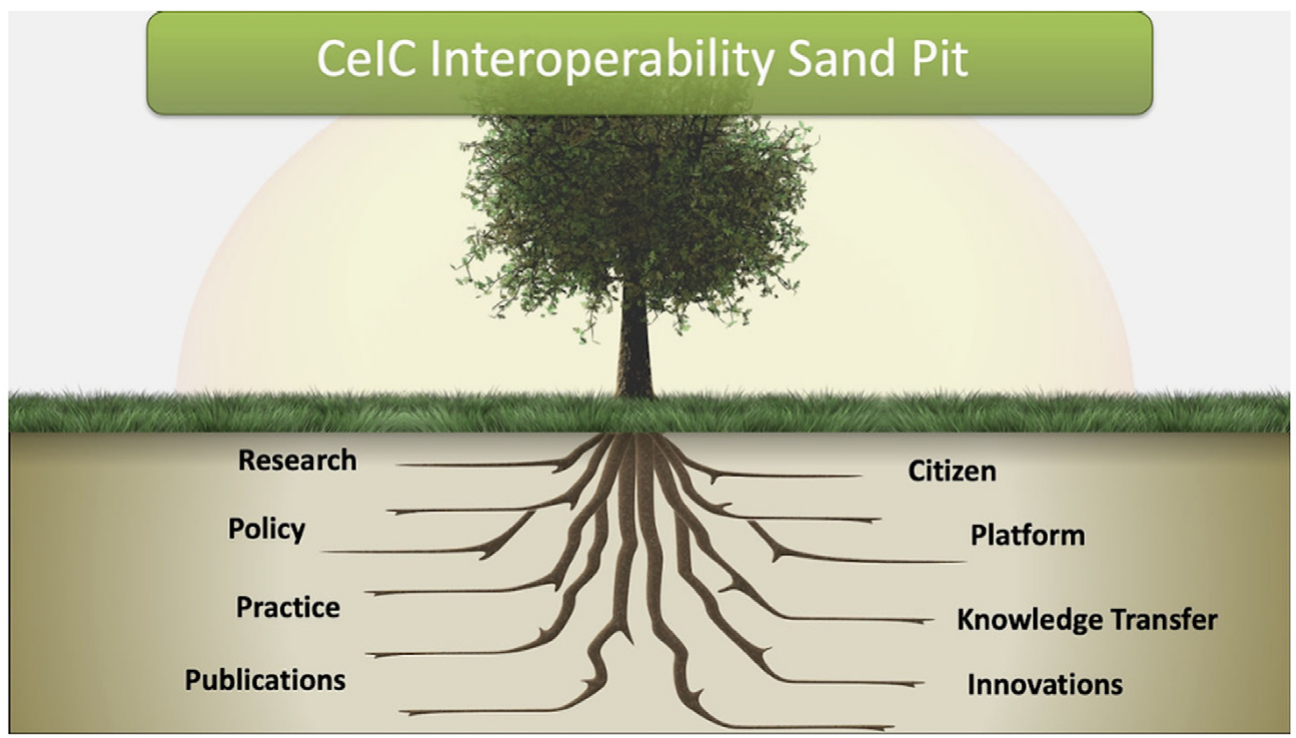
Home page for the *Centre for eIntegrated Care* (CeIC) interoperability sand pit.

The key message of the resources are to offer focused and targeted material and learning activities to explain the process of interoperability in the context of health and social care. The aim of the resource was to assist in advancing sustainable eHealth tools and solutions through knowledge transfer on interoperability. The resource would emphasize the importance of standards, open source infrastructure and stress the importance of interoperability as a core building block in good system design promoting resilience and sustainability for digital health. The associated online platform to support the MC therefore adopted a visual representation of a root tree structure as presented in Figure 1. This diagram provides a screen shot of the landing home page for the MC online resources illustrating a visualization of how each of the represented sections forms a root to anchor health information systems design architecture with interoperability theory practice and associated processes.

Eight sections were identified for inclusion in the online resources, containing a suite of digital assets often containing single files, guidance and raw media assets.

**Figure 2.**
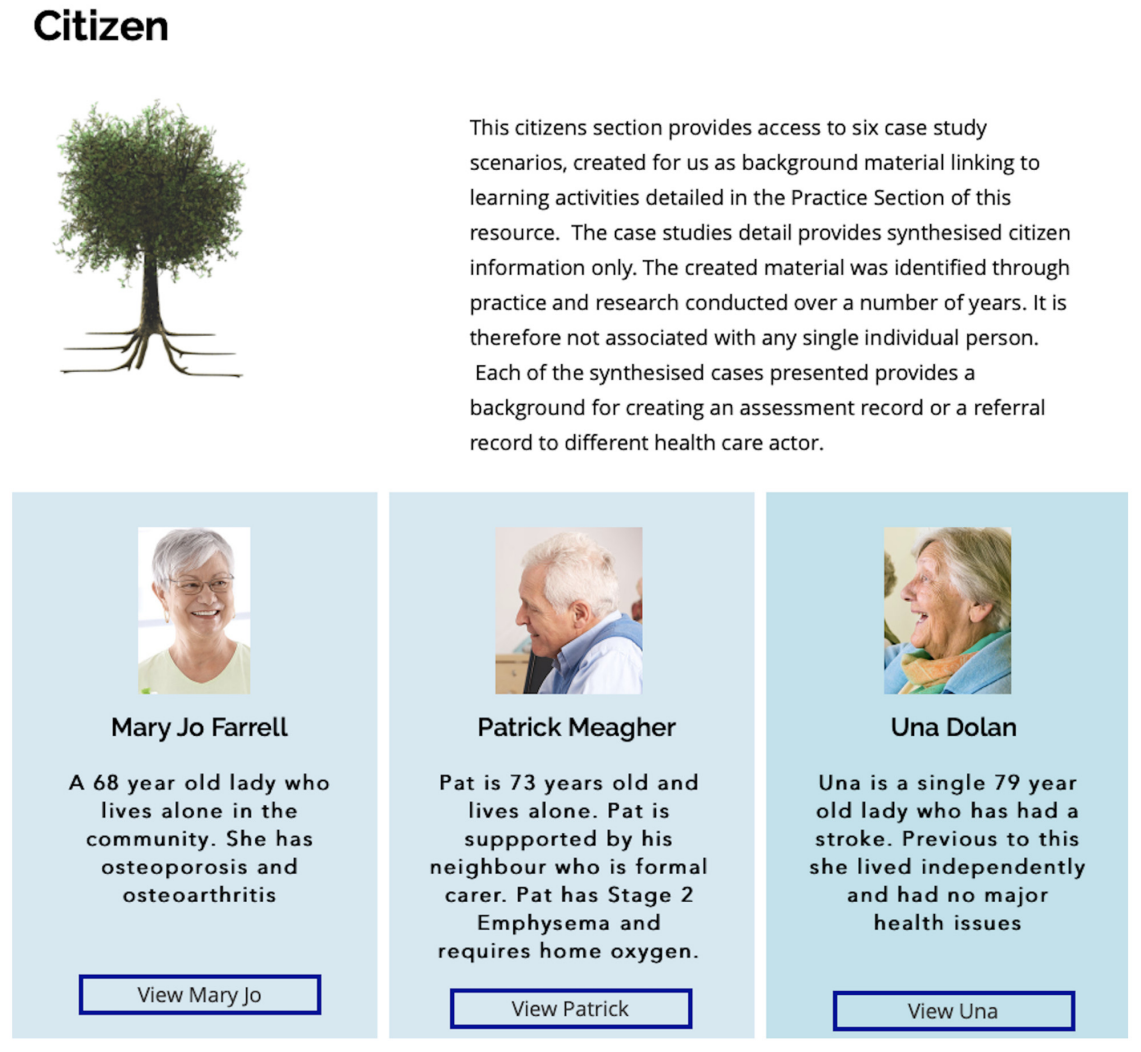
Citizen section.

***3.1.1 Citizen.*** The Citizen section (See Figure 2) presents six detailed synthesised case studies with associated material from a community or community residential service background. Each citizen scenario includes a linked brief biography, health maintenance detail with health management issues and some background on the citizen’s routine activity and communications. Learning hooks for development of material were also included such as the citizen stories designed to identify and document a reoccurring issue in health and social care which could inform lessons for role play and scenarios building. For example cases included present citizens with chronic diseases such as emphysema or rheumatoid arthritis. The citizen cases are all living in place and require services to support integrated self-management support action plans. The citizen section acts as a primer for creation of use cases in later sections of the resource under the practice section and additional master classes in the MC.

**Figure 3.**
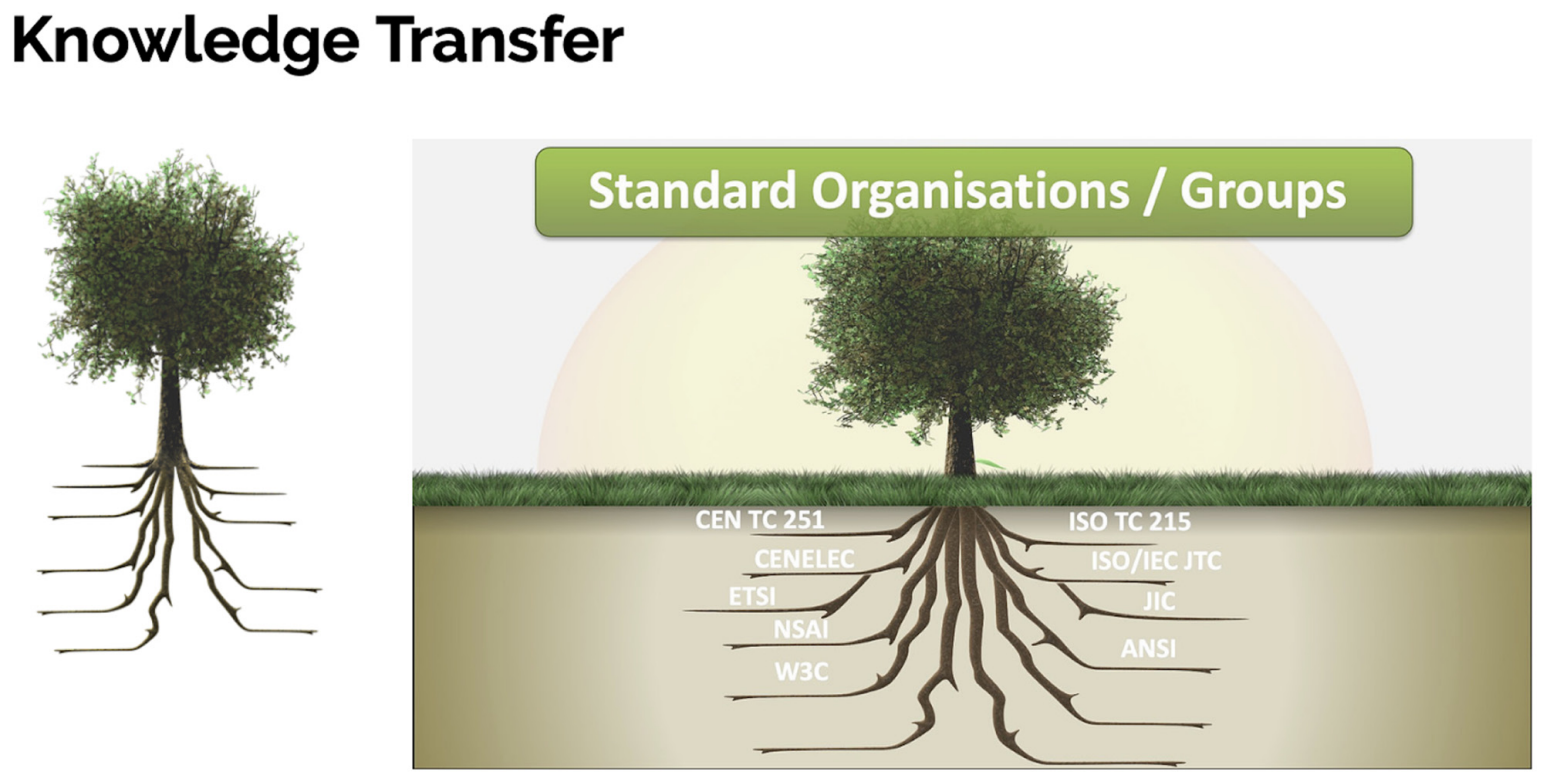
Knowledge Transfer section.

***3.1.2 Knowledge Transfer.*** At the heart of Knowledge Transfer (see Figure 3) in the domain of health informatics is the world of standardisation. Standards work focuses on building consensus on practical services software products and processes to deliver quality and impact positively on outcomes. In this Knowledge Transfer section we include the key organisations which are pivotal in shaping and designing the future systems and services in the domain of health informatics globally. We also draw extensively on the work of experts in the field of interoperability and include a short presentation explaining how systems designers progress from models of use to models of meaning ^7, 8, 15^.

**Figure 4.**
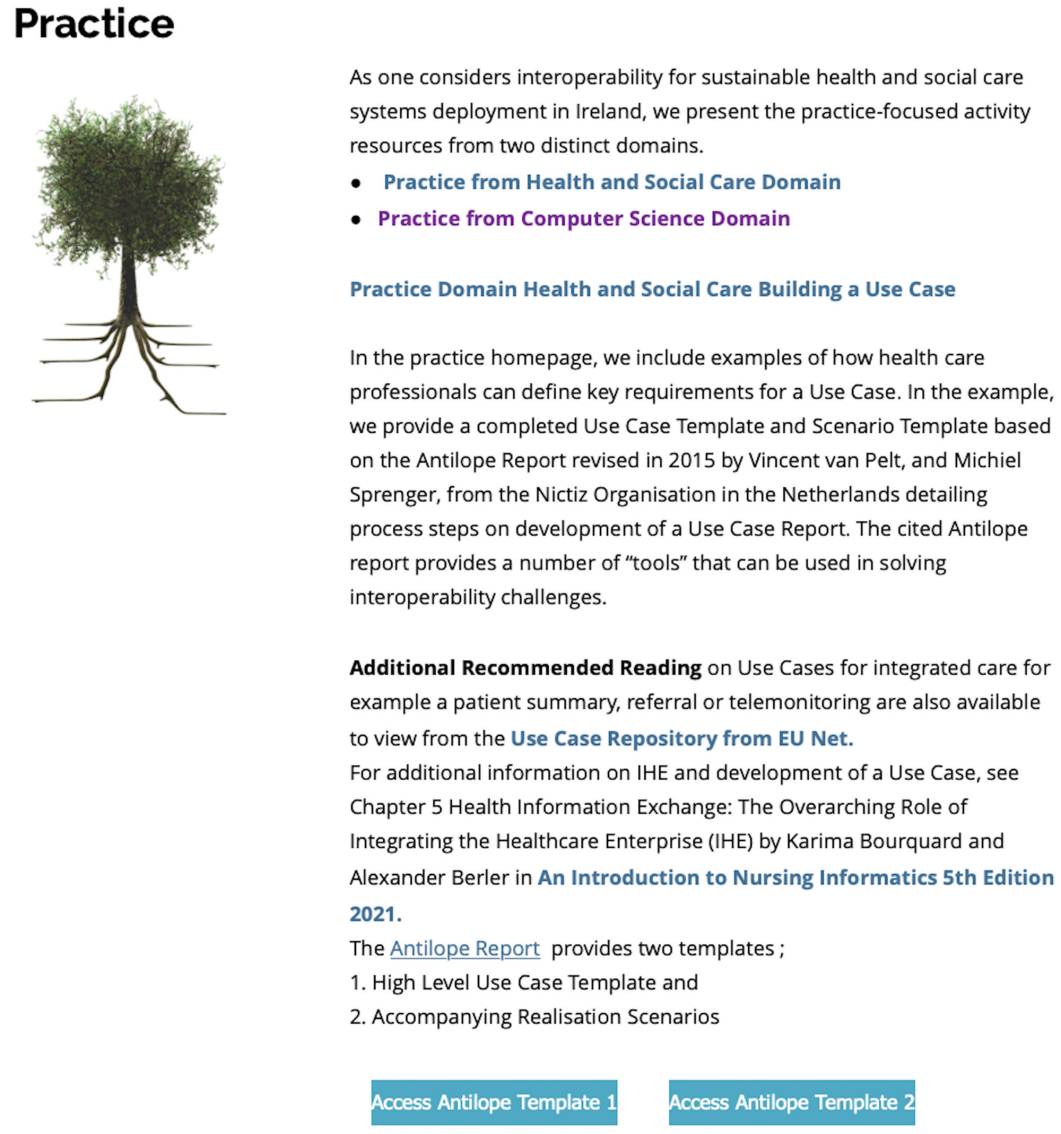
Practice section.

***3.1.3 Practice.*** The Practice section (see Figure 4) is created to assist health care professionals to understand the multifaceted nature of the process of design for interoperability to advance integration moving from designing use cases with domain experts from the health and social care domain to creating schemas ontologies and GraphDB ^30^ visualizations through a guidance resource describing the activity involved in a series of action steps. See online link for the details https://sites.google.com/dcu.ie/csdm/. Prototype for Clinical assessment which is mentioned under in this section i.e. *Practice from Computer Science Domain* can be access via link https://arcg.is/CXamK

**Figure 5.**
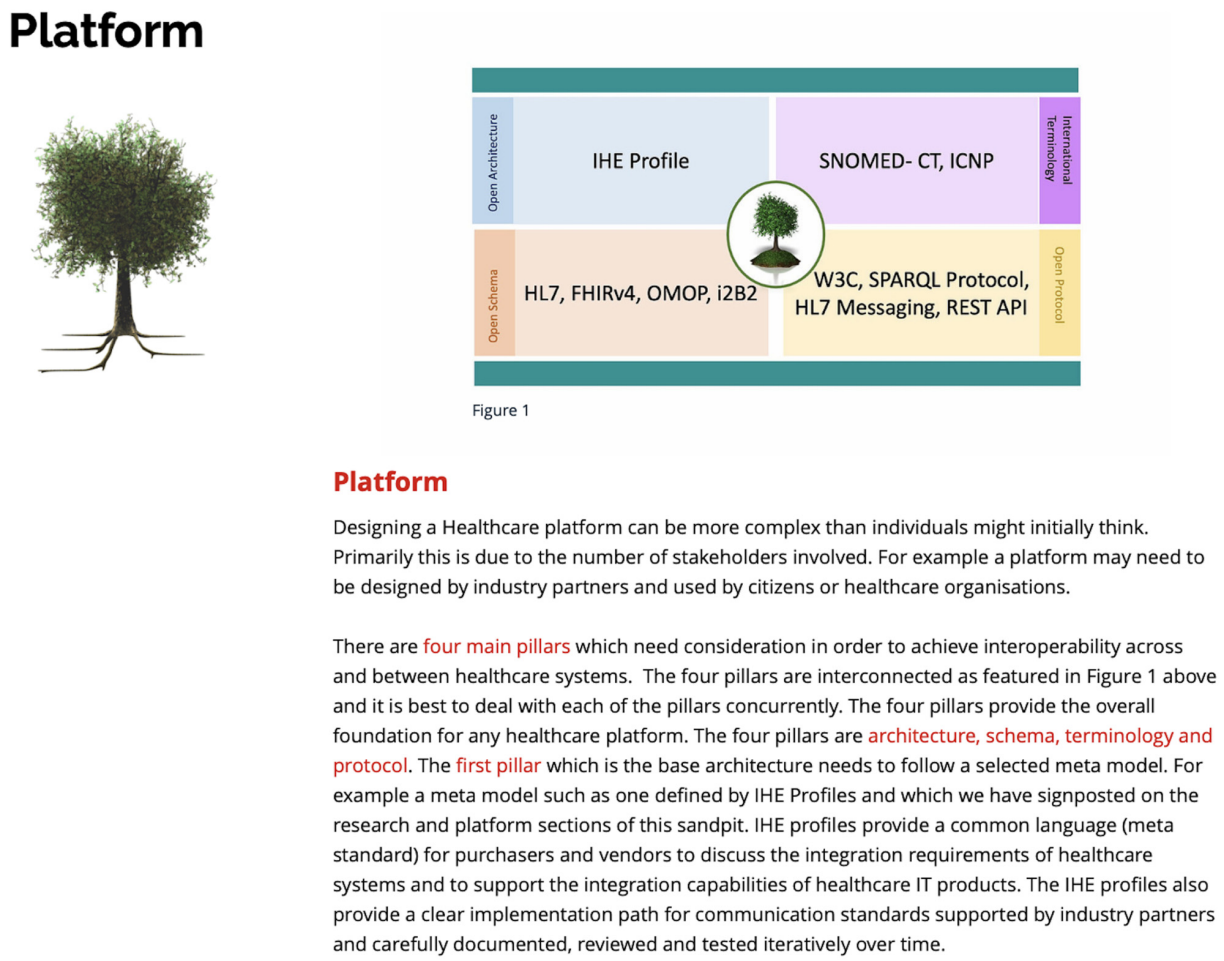
Platform section.

***3.1.4 Platform.*** Following on from the Practice section and some associated learning activity exercises, the Platform section (see Figure 5) provides a beta version of the CSDM demonstrator from, the Elite S Marie Curie fellowship, previously mentioned in Section 2.2 ^14, 21^. The platform section is designed to introduce the participants to relevant standard specification for schema and machine to machine communication. Examples in the demonstrator includes Fast Healthcare Interoperability Resources (FHIR) based application with supporting applicable ISO Standards and specifications ^15, 31^. Full documentation of Formal Ontology of Continuity of care can be access via link https://subhashishhh.github.io/contsysDoc/ and this version of the ontology schema can be cite as *doi:10.5281/zenodo.5419092*.

**Figure 6.**
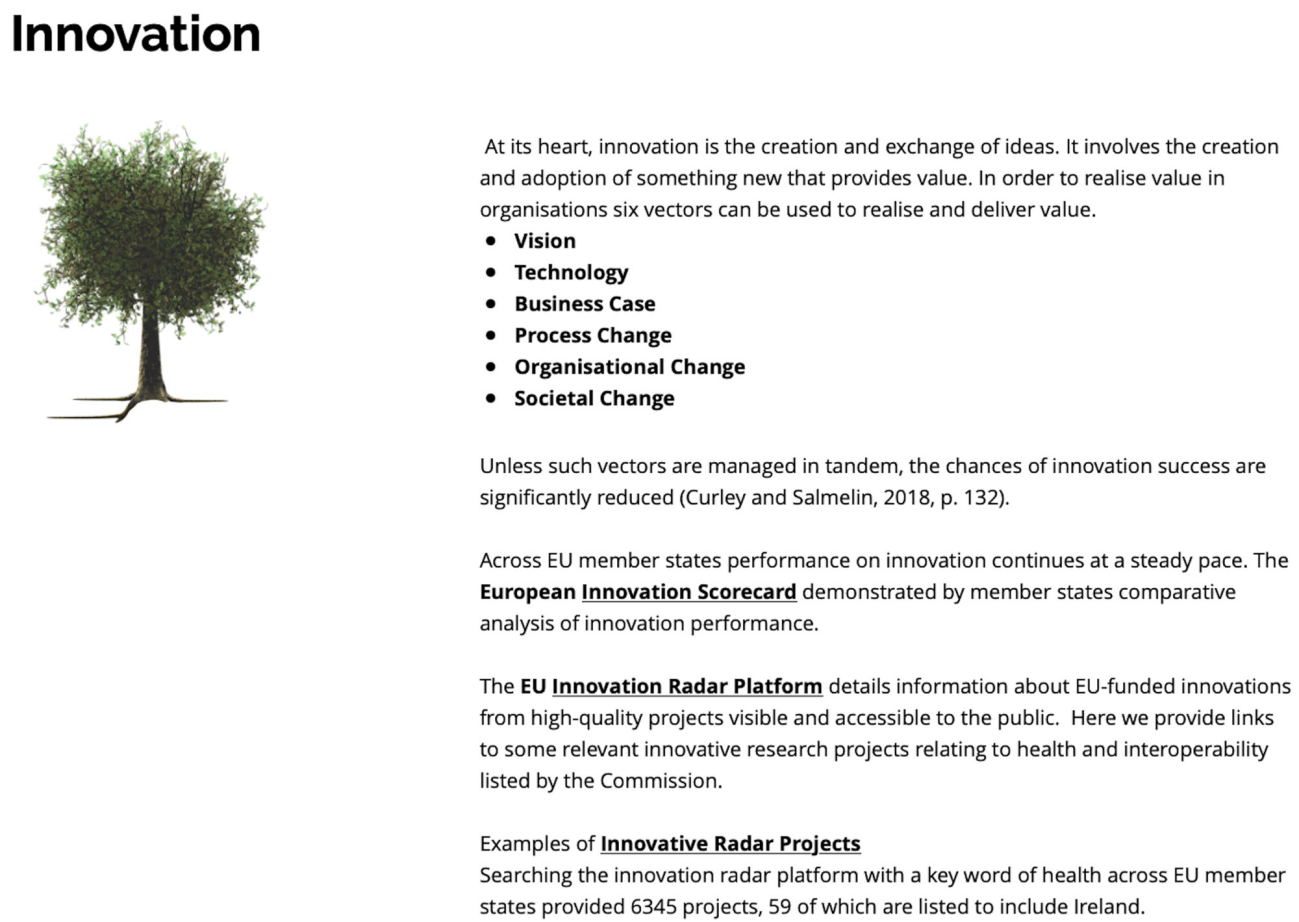
Innovation Section.

***3.1.5 Innovation.*** Under the Innovation section (see Figure 6), the user is introduced to key strategic activities published by the European Commission. For example the European Interoperability Framework and associated resources are listed. In addition the innovation section offers a suite of projects selected to represent the EU agenda for driving digital through research. Recent publications such as the Research European Observatory for ICT Standardisation which promotes the development and participation of citizens to engage with a European wide ecosystem are also included ^32^.

**Figure 7.**
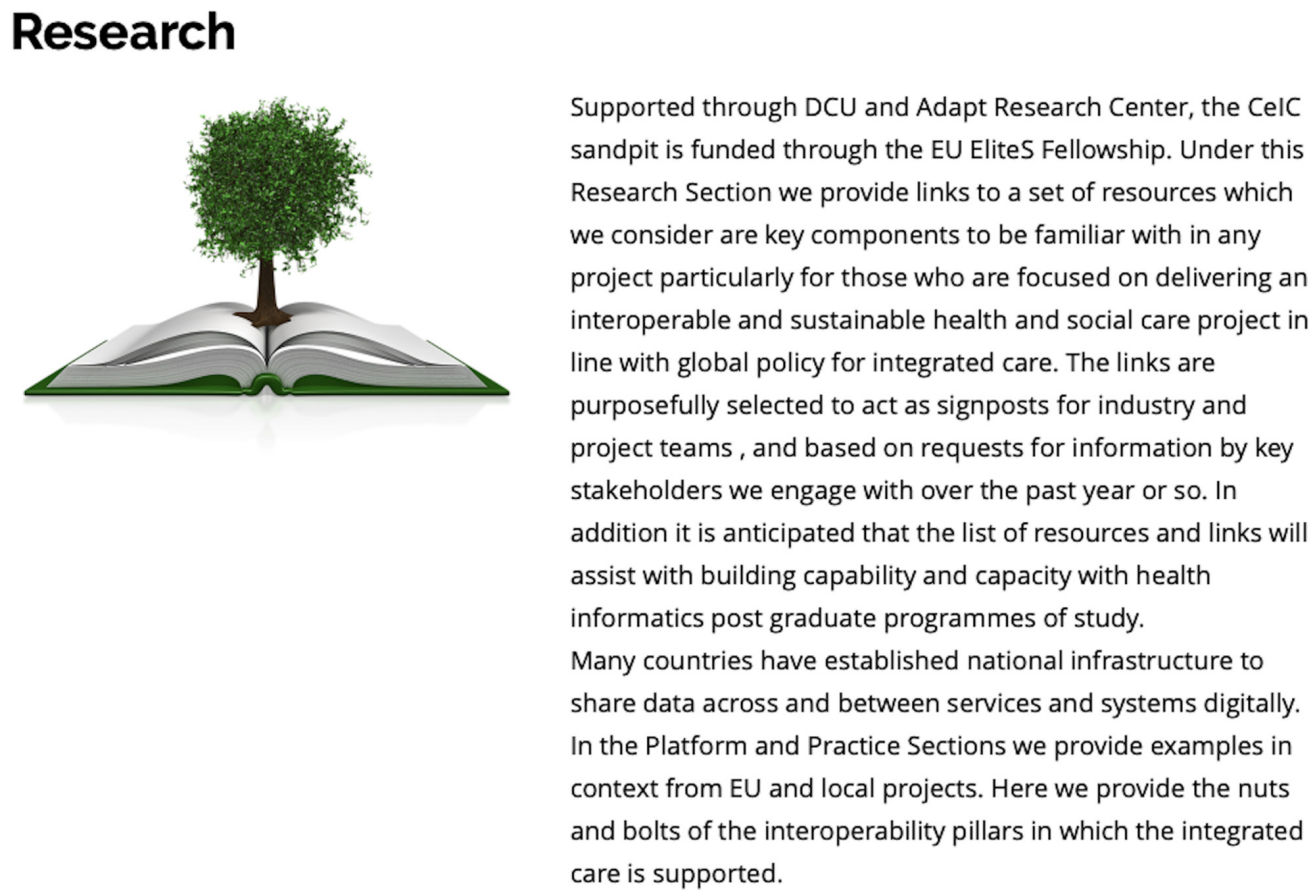
Research section.

***3.1.6 Research.*** The Research section (see Figure 7) offers a dedicated set of resources purposefully collected by the authors to be reviewed over time by the participants engaged in the MC. For example Open Standards links and metadata standard web pages are flagged to illustrate the nuts and bolts of interoperability. Key infrastructure used widely in deployment of shared health records such as Health Level Seven (HL7) and the IHE IT Infrastructure (ITI) Technical Framework (IHE ITI-TF) Integration Profiles are included for review. The research section also includes a glossary of terms which will evolve over time as the resource is used in training as part of the MC programme ^33, 34^.

**Figure 8.**
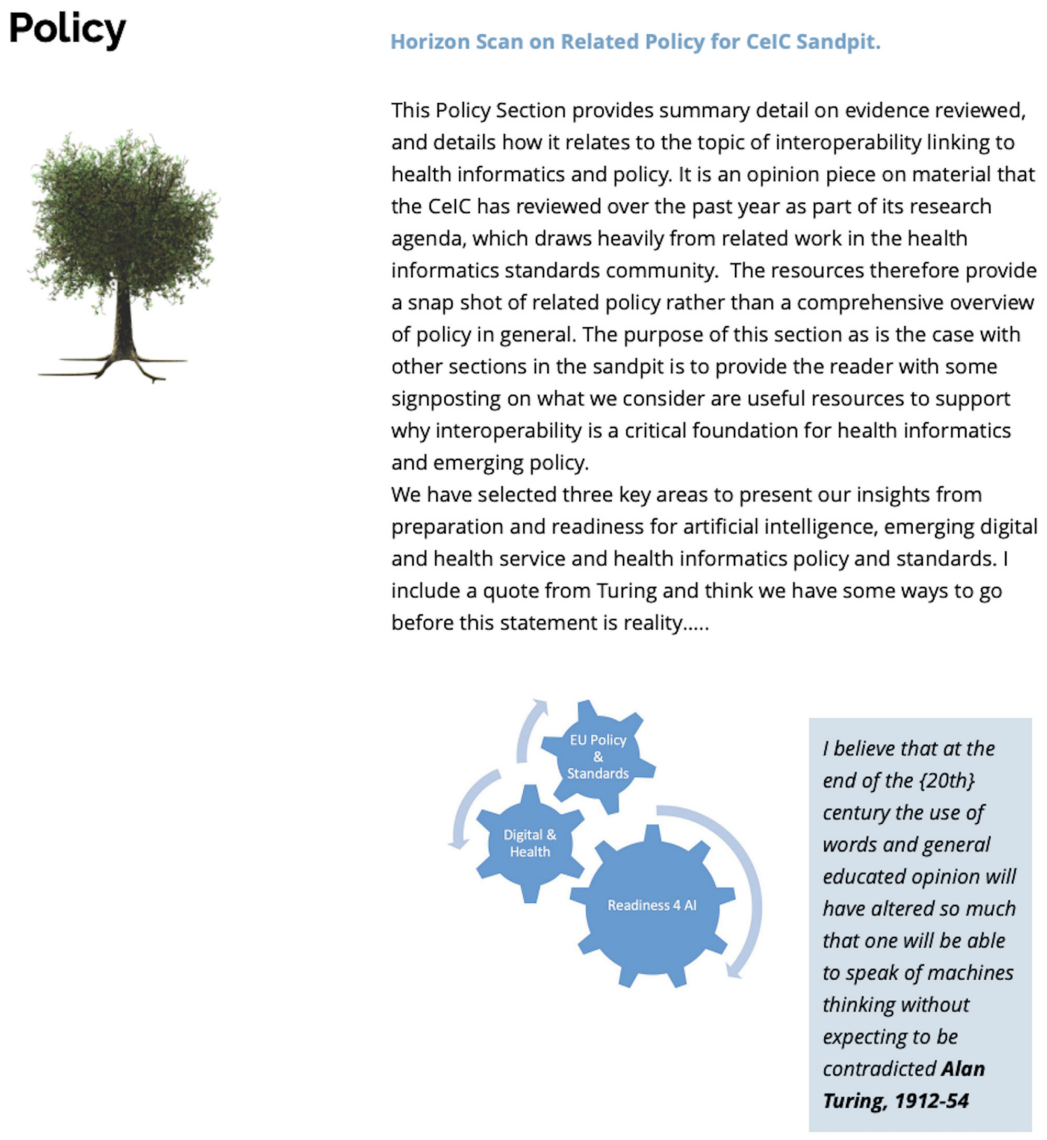
Policy Section.

***3.1.7 Policy.*** The Policy section (see Figure 8) provides an opinion piece on material reviewed by the *Centre for eIntegrated Care* (CeIC) over the past year. This policy section draws heavily from related work in the health informatics standards community. The resources therefore provide a discrete snapshot of related policy to the topic of interoperability rather than a comprehensive overview of policy relating to the topic in general. The purpose of this section as with other sections in the MC resources, is to provide the reader with some signposting on relevant resources to support why interoperability is a critical foundation block for emerging policy and to stimulate discussion and reflection in related discussion fora.

**Figure 9.**
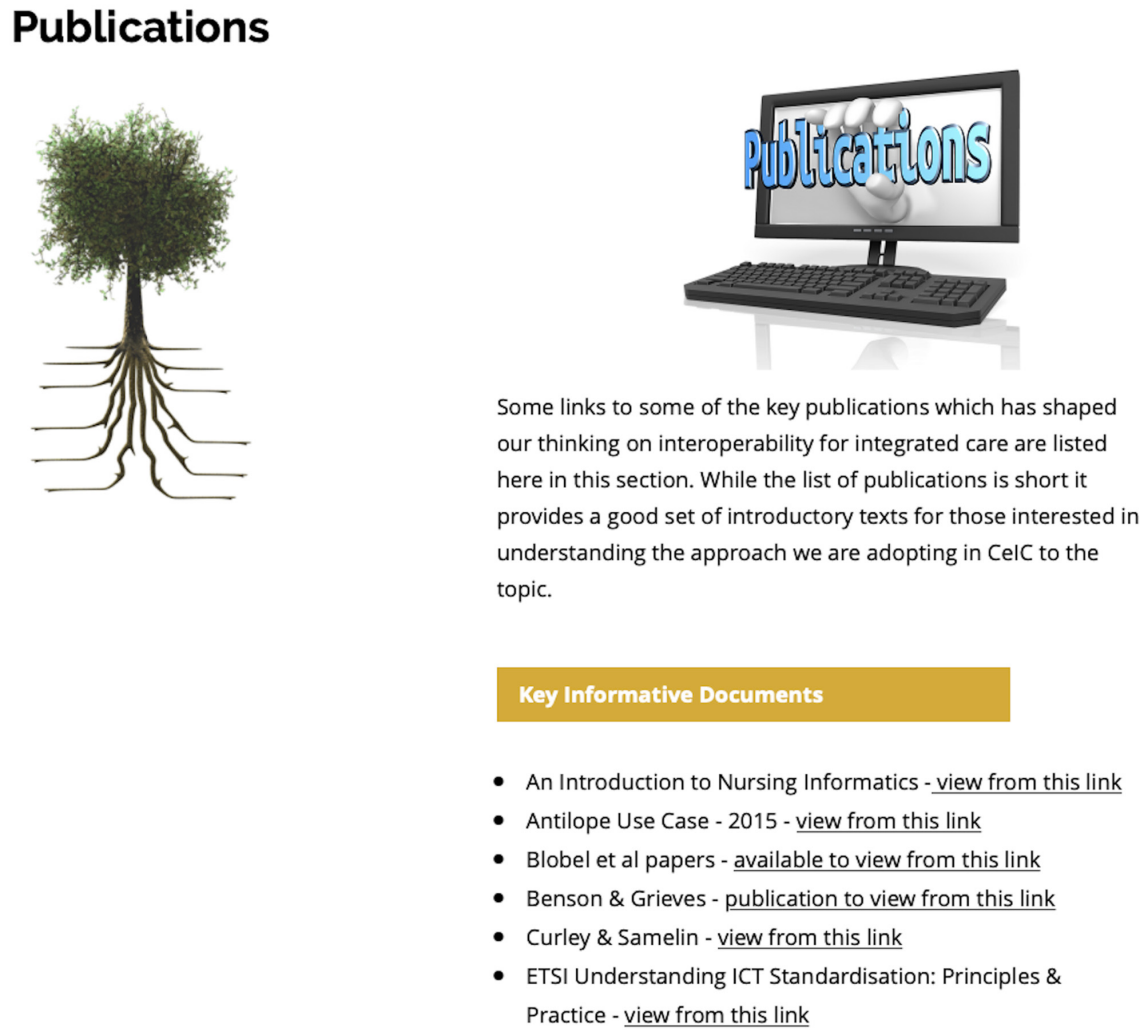
Publication section.

***3.1.8 Publications.*** The Publications section (see Figure 9) links to some key authors and resources which has shaped the authors thinking on interoperability research for integrated care. While the list of publications is short, it is presented as a set of introductory texts for those interested in understanding the approach adopted in the *Centre for eIntegrated Care* CeIC to the topic.

## 4 Discussion

In the context of health and social care, there is a growing need for domain experts to understand the importance of, and theoretical underpinnings on the topic of interoperability. For clinicians and those interested in the topic of interoperability with limited knowledge on informatics, there is a need to create targeted micro credential (MC) for scale up and spread of competency in this important field of health and computer science. Developing an MC which offers foundational signposting to material on the topic through context specific configurations underpinned by interdisciplinary practice is considered a worthwhile initiative to invest time in. Through focused collaboration the topic of interoperability is identified as a foundation stone to deliver on the digital health agenda. Technical reports related papers and specifications published by the International Organization for Standardization (ISO) community stress the importance of good quality data, reporting that such emerging technologies will use data in Systems of Interest (SoI) as a basis for decision making and predictive modelling ^16, 35^. The design brief was identified to create a focused innovative resource which offers scaffolds for learning and which can be used as part of a MC for interoperability. The initial collaboration with stakeholders learning and interested in this topic suggests that the resources created to date provide an initial platform to support the advancement of an MC for interoperability. As educators, the academic community has a role to play publishing research and innovation through open science ^21, 36^.

The need for understanding the importance of interoperability has never been greater as research teams strive to integrate specifications across and between different domains and sub domains to advance digitalisation in care delivery.

In health and social care services, the clinician has a key role in sense checking the clinical information, the associated data, and the relationships there in to optimise safety and minimise risk. At an enterprise level, new systems deployment such as artificial intelligence (AI) and machine learning (ML) for example can collect, combine, and process data from different sources providing capacity to conduct one or more given tasks for care delivery but the topic is complex and the risk for data breaches and malicious privacy violations has never been greater ^37^. At its core is the need for AI to have access to quality orientated and clean data to apply knowledge from defined domain information models is recognised ^18^. To move beyond siloed systems, a European Strategy for Data published in 2020 identifies a single European Data Space as a priority where data can flow within the EU and across sectors ^38^. Designing strategies for instruction or providing targeted resources such as the MC for interoperability may assist in some small way to inform those learning about interoperability to understand its significance for scale up and spread of resilient and sustainable solutions to support a quality orientated digital health service. Next steps for this MC is to pilot test it in the next academic year (2021–22). In the interest of sharing knowledge on development processes and linking academics and stakeholders together through established ecosystems this paper provides a summary of progress and deliverables to date.

By focused interaction among the producers and users of research, tailoring information to different target audiences so that interventions and associated resources are used more widely is considered important to accelerate and modernise pubic health services ^39^. Such initiatives can act as institutional bridges between researchers, decision-makers and communities. More than ever before, countries need to counter misinformation and rapidly mobilise the best available evidence, and present it in user friendly ways to decision-makers. Future directions for this interoperability resource include progressing the development of MC as part of the European Consortium of Innovative Universities (ECIU) initiative by piloting it with colleagues in the US, Italy and Ireland ^6, 40^. Universities increasingly will need to play several roles in their communities, and one of their key functions is to support and drive regional, social and community developments. With an eye to the future, it is important for universities to invest in innovative activities in order to have a better chance of making a sustainable impact on service improvement in the medium to long term. Capitalising on existing funded projects and exploiting internal knowledge and resources through shared scholarship facilities using Open Innovation 2.0 (OI2.0) methods is one approach to avoid wheel reinvention ^41, 42^. We consider such initiatives may be small in size, but none the less we consider that they are important to rapidly drive sustainable transformation and forge connections with likeminded people for the future. We conclude with recent quote from World Health Organisation (WHO) Director Dr Tedros Adhanom Ghebreyesus who suggests that *"We’re not just fighting an epidemic; we’re fighting an infodemic"* – lets try and break the cycle of heterogeneity which impedes data analysis by advancing interoperability.

## Data availability

No data are associated with this article.

## Software availability

- Source code available from: https://github.com/subhashishhh/contsysDoc/tree/v1.4.0

- Archived source code at time of publication: https://doi.org/10.5281/zenodo.5419092 ^29^

- License: Data are available under the terms of the Creative Commons Zero "No rights reserved" data waiver (CC0 1.0 Public domain dedication).
