# Supplementary material for: A micro credential for interoperability
Source: Open Res Eur. 2021 Sep 15;1:109. [Version 1] doi: 10.12688/openreseurope.14083.1 (PMC10446011; doi:10.12688/openreseurope.14083.1)
Supplement: Peer review history

# Peer review: Reviewer response for version 1

**DOI:** 10.21956/openreseurope.15181.r28442

**Published:** 2022-02-01

**Authors:** Dorota Kilanska

**Affiliations:** 1 Department of Health System Development, Medical University of Lodz, Lodz, Poland

This innovative approach to the possibility of obtaining data for the creation of open science is worth disseminating. 

 All process steps have been defined and well described based on a clear methodology. The attached images allow you to illustrate the efforts of the creators to create a tool that will allow you to achieve the intended goal.

 The publication shows not only the methodology of the approach to solving the problem, but also shows the importance of involving universities in the implementation of innovations that will benefit society.

Is the case presented with sufficient detail to be useful for teaching or other practitioners?

Yes

Is the work clearly and accurately presented and does it cite the current literature?

Yes

If applicable, is the statistical analysis and its interpretation appropriate?

Yes

Are all the source data underlying the results available to ensure full reproducibility?

Yes

Are the conclusions drawn adequately supported by the results?

Yes

Is the background of the case’s history and progression described in sufficient detail?

Yes

Reviewer Expertise:

IT, nursing, coordinated care, cost-effectiveness

I confirm that I have read this submission and believe that I have an appropriate level of expertise to confirm that it is of an acceptable scientific standard.

# Peer review: Reviewer response for version 1

**DOI:** 10.21956/openreseurope.15181.r27965

**Published:** 2021-11-02

**Authors:** Paul De Raeve

**Affiliations:** 1 European Federation of Nurses Associations, Brussels, Belgium

The article reads very easy and is key to address the interoperability challenges in the EU. The strength of the article is the accessibility to any reader, including HCP.

 Related to "The breadth and depth of literature relating to the levels of engagement on interoperability ... significant." perhaps consider mentioning the October 2021 Policy Paper on Interoperability developed by EFN and HOPE within the EU eHealth Stakeholder Group. This working paper will be published soon but is available on request to the author.

 2 typos:

 topic ofinteroperability - processof engagement

 The strength of this article are the detailed description of the "Eight sections", with key focus on “citizens”, “platform” and “knowledge transfer” which is crucial for the EU citizens. Furthermore the article's strengths lies within the section “policy” and “clinical practice”, what will be key for 3 million nurses in the EU.

 The graphics used to explain the text is very welcomed as it helps understand the content as interoperability can be something very technical. This makes this article so unique.

 It would be interesting to have in the discussion a reference to gender! Furthermore, the article is addressed to all HCP, but I am wondering if there is a difference in approach between nurses and doctors, which also links to gender!

Is the case presented with sufficient detail to be useful for teaching or other practitioners?

Yes

Is the work clearly and accurately presented and does it cite the current literature?

Yes

If applicable, is the statistical analysis and its interpretation appropriate?

Not applicable

Are all the source data underlying the results available to ensure full reproducibility?

Yes

Are the conclusions drawn adequately supported by the results?

Yes

Is the background of the case’s history and progression described in sufficient detail?

Yes

Reviewer Expertise:

EU Expert on Digitalisation, Continuity of Care, EU Policy, AI, ....

I confirm that I have read this submission and believe that I have an appropriate level of expertise to confirm that it is of an acceptable scientific standard.
